# Gallbladder Mucus Plug Mimicking Ascaris Worm: An Ambiguous Cause of Biliary Colic

**DOI:** 10.1155/2017/7167934

**Published:** 2017-11-26

**Authors:** Salah Termos, Mohammad Alali, Majd Alkabbani, Abdullah AlDuwaisan, Ahmad Alsaleh, Khalifa Alyatama, Hussein Hayati

**Affiliations:** Hepatobiliary and Transplant Unit, Department of Surgery, Al-Amiri Hospital, Kuwait City, Kuwait

## Abstract

Biliary colic is a visceral pain caused by attempts of the gallbladder or bile duct to overcome the obstruction in the cystic duct or ampulla of Vater. Obstruction can be due to different etiologies such as stone, mass, worm, and rarely by mucus plug. We report the case of a 31-year-old gentleman who presented with recurrent biliary colic and weight loss. Work-up showed linear calcifications in the gallbladder extending to the common bile duct suggesting hepatobiliary ascariasis. Further investigations including stool analysis, upper endoscopy, endoscopic ultrasonography (EUS), and endoscopic retrograde cholangiopancreatography (ERCP) did not support our provisional diagnosis. Laparoscopic cholecystectomy was performed. Histopathological finding was grossly ambiguous; a rope-like mucus plug resembling ascaris worm was noted. The patient's condition improved instantly after the procedure. To our knowledge, we are reporting the first case in the English literature describing this unique entity of symptomatic gallbladder disease to increase awareness and improve its management.

## 1. Introduction

Biliary colic is usually caused by the gallbladder contraction in response to hormonal or neural stimulation, forcing a stone or possibly sludge and rarely a worm or a mucus plug against the gallbladder outlet or cystic duct opening, leading to increased intravesicular pressure [1]. We describe an unusual cause of obstruction due to a mucus plug resembling a worm that was diagnosed and managed as biliary ascaris.

## 2. Case Presentation

**Figure 1:**
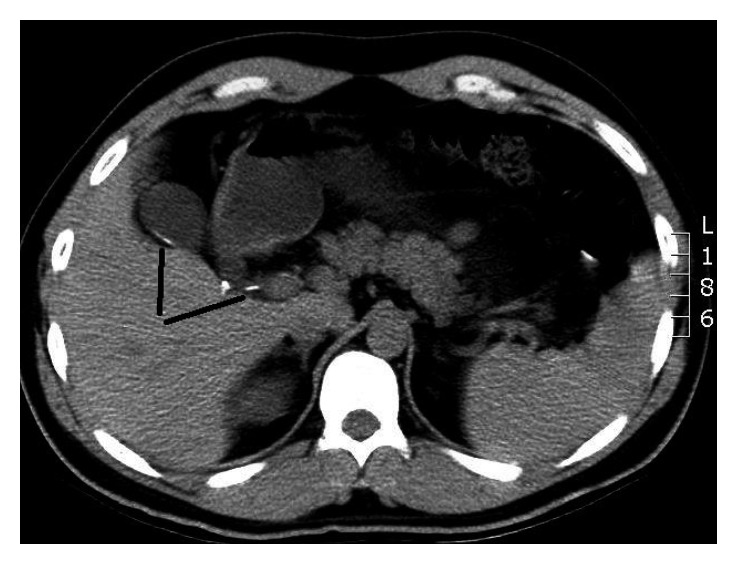
CT abdomen: linear calcification extending from the gallbladder through the cystic duct (dashes).

**Figure 2:**
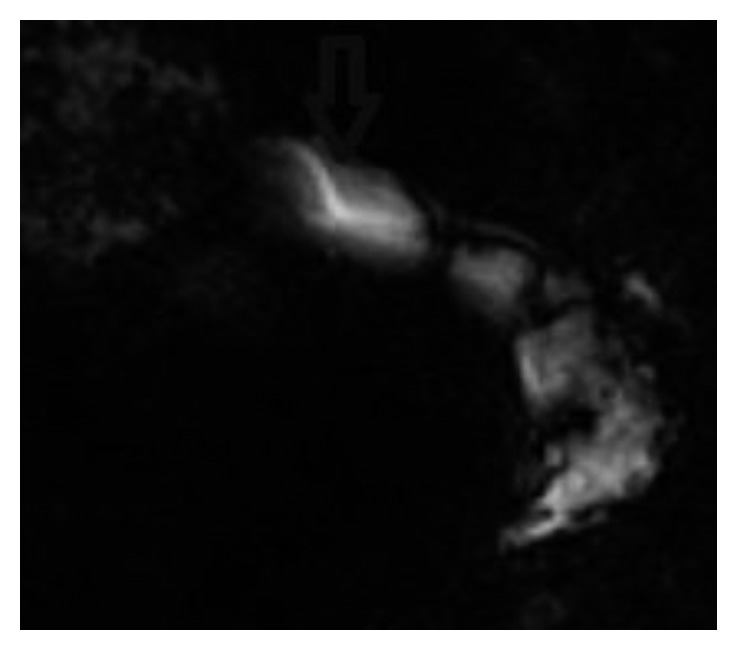
Magnetic resonance cholangiopancreatography (MRCP) demonstrating the 3-line sign typical for ascaris worm (arrow).

In our manuscript, we report the case of a 31-year-old Syrian gentleman, who was previously healthy, presenting to the emergency room with repeated bouts of biliary colic. His pain was frequent, lasting for about two hours, and alleviated with pain killers and antispasmodics. The symptoms were exacerbated by heavy meals. He also reported a significant weight loss of 10 kilograms in two months. His vital signs and laboratory tests were all within the normal ranges. Ultrasound abdomen showed a contracted gallbladder with intraluminal calcification. Initial work-up began with an enhanced computed tomography (CT) scan, which revealed linear dense calcifications within the gallbladder extending into the cystic duct to the junction of the common bile duct (Figure 1). Further investigations were conducted. Stool analysis did not detect any egg or parasite. Upper endoscopy and ERCP showed the absence of worms in the duodenum or inside the biliary tree, respectively. The patient did not note any family history of biliary cancers. Tumor markers were within the normal ranges, and MRCP showed a 3-line sign and was unremarkable for any other abnormalities (Figure 2). EUS elicited a hyperechoic calcified linear structure measuring 4 × 0.5 cm. We proceeded for laparoscopic cholecystectomy. Intraoperatively, the liver looked normal and the gallbladder appeared contracted with no sign of inflammation or malignancy. It was easily removed, and frozen section of the cystic duct was negative for any pathology.

**Figure 3:**
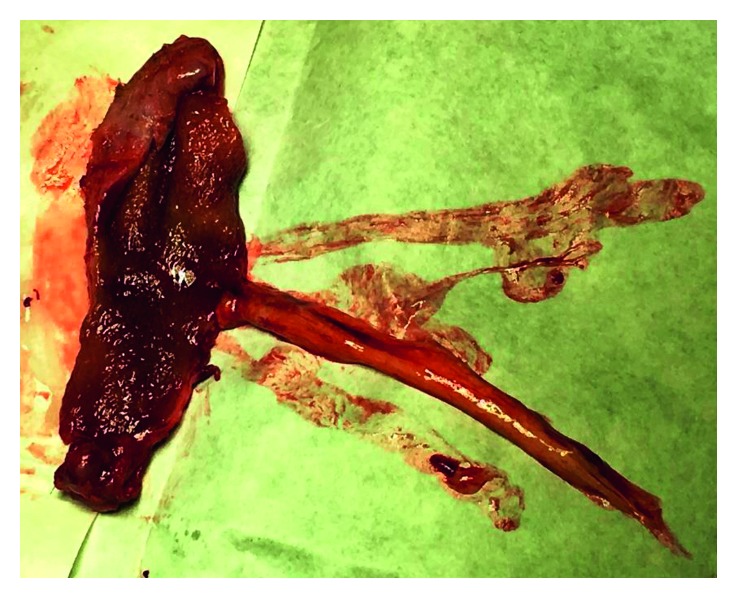
Gross specimen of the gallbladder with a mucus plug mimicking a worm.

**Figure 4:**
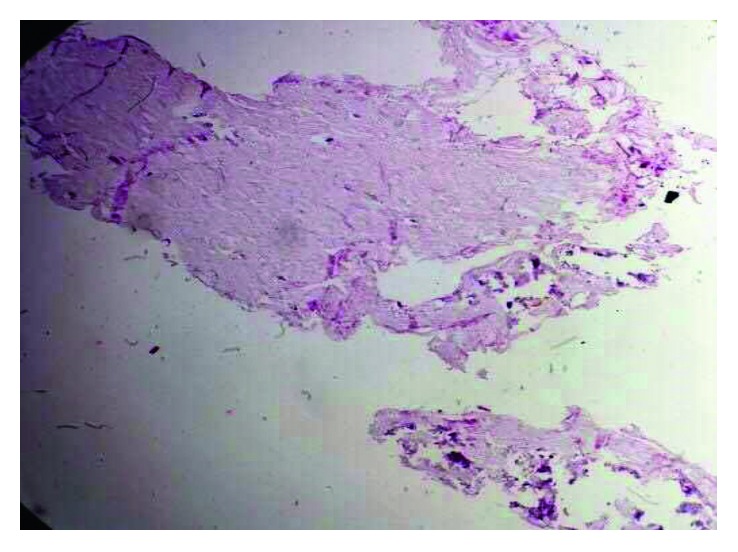
Microscopic examination of the tubular structure confirming a thick mucus content and absence of parasitic infestation.

Gallbladder gross examination was suggestive of a parasitic worm with some sludge (Figure 3). However, the definitive histopathological study revealed a chronic cholecystitis with thick mucus content and no evidence of a parasitic infection, worm, larva, or ova (Figure 4). The mucus plug in the gallbladder was seen wrapped in a tubular fashion mimicking ascaris. The patient had an uneventful postoperative hospital stay. He is currently asymptomatic on follow-up one year after his surgery and regained his weight.

## 3. Discussion

Biliary colic can present with different manifestations and due to many causes. Our patient is a young gentleman who suffered from typical colicky right upper quadrant pain, moderate in severity over a period of six months. It was associated with weight loss, which was likely due to fear of triggering the pain, and in part due to anxiety from the extensive work-up and the unusual etiology of his condition. Our differential diagnosis leant towards a parasitic disease based on results of his imaging studies. His stool analysis, however, was negative for any egg or helminth, and upper endoscopy did not show any sign of parasites or abnormalities.

Helminthic infestation occurs most commonly with *Ascaris lumbricoides*, *Clonorchis sinensis*, *Opisthorchis felineus*, and *Fasciola hepatica* [2]. Ascaris is a roundworm and is one of the most prevalent helminthic hepatobiliary parasites in humans worldwide. Fortunately, it only infrequently produces symptomatic disease. *Ascaris lumbricoides* normally reside in the jejunum but are actively motile and can invade the papilla and thus migrate into the biliary system causing biliary obstruction with a variety of hepatobiliary complications. Biliary colic (56%), acute cholangitis (24%), acute cholecystitis (13%), acute pancreatitis (6%), and rarely hepatic abscess have all been reported as complications of this parasite [3].

Radiographic imaging of biliary ascariasis is usually pathognomonic. On ultrasound, we may find long, linear, parallel echogenic structures without acoustic shadowing or the presence of “four-line sign.” Nonshadowing echogenic strips with a central anechoic tube representing the parasite's digestive tract, indicative of the worm and its intestines, may also be seen [4]. On CT scan, a nonenhancing coiled tubular structure of soft tissue density with specks of curvilinear disk-like lesion or linear calcification may be seen. In other situations, such ovoid lesions may represent a neoplasm in the absence of clinical suspicion of ascaris.

MR cholangiogram may show intraductal worms as a linear low-intensity filling defect in the bile ducts. The “three-line sign” appears to be a characteristic sign of biliary ascariasis on 3D magnetic resonance cholangiopancreatography (MRCP) [5]. ERCP is highly sensitive in detecting parasites in the biliary and pancreatic ducts. Worms can be seen in the duodenum and very often across the ampulla of Vater during endoscopy [6]. Cholangiographic findings of the ascaris worm during ERCP include long, smooth, linear filling defects with tapering ends as well as parallel, smooth filling defects, curves, and loops crossing the hepatic ducts transversely and dilation of the bile ducts (usually the common bile duct).

Our case is ambiguous due to the paucity of these radiological signs in such a common medical illness. It is well known that ill-defined lesions may simulate the presence of a neoplasm in the absence of clinical suspicion of ascaris. We investigated the patient thoroughly for malignancy using laboratory and radiological testing, in addition to intraoperative frozen section analysis which was also negative for malignancy. Our preoperative and postoperative diagnosis favored gallbladder ascariasis as the patient was from Syria, as the condition is endemic in this region [7]. However, the microscopic examination surprisingly excluded our provisional diagnosis.

According to the literature, similar conditions may occur if the cystic duct is atretic or stenotic due to inspissated mucus or mucosal hyperplasia. This usually leads to a contracted gallbladder with some sludge. Our patient denied any past antecedent of jaundice or family history of cystic fibrosis or any metabolic disease [8]. The mechanism of mucus secretion remains unclear, and the secretory granules in the chief cell of the gallbladder epithelium were microscopically observed to secrete mucus by a mechanism similar to that of merocrine secretion [9]. Mucus glycoprotein overproduction has been investigated as an important factor during the formation of gallbladder stones. Experimental results note that the increase in the cholesterol content of bile can stimulate gallbladder mucus hypersecretion [10].

The presence of thick mucus sludge in the gallbladder can be encountered in conditions associated with endocrine disorders, such as hypothyroidism, and in mucoceles. A mucocele refers to an overdistended gallbladder filled with mucoid fluid. It results from outlet obstruction of the gallbladder in the neck of the gallbladder or in the cystic duct [11]. Roundworms can cause this due to bile duct obstruction; however, our case presented with a clinical picture of acute on the top of chronic cholecystitis with no evidence of parasitic infection or mucocele. A mucus plug etiology in a case such as ours is unusual. It is known that mucus formation can be a result of lithogenic bile, and it is associated with biliary sludge. In a literature review, we did not find any similar condition that had been described in the past. Presence of contracted gallbladder and absence of plentiful Rokitansky–Aschoff sinuses preclude the diagnosis of mucocele [12].

## 4. Conclusion

Biliary colic is a common clinical manifestation that occurs due to various causes. Linear ground-glass opacification inside the gallbladder usually indicates hepatobiliary ascariasis. Absence of objective findings may suggest different pathologies. A rope-like mucus plug in our case was a unique entity, presented with symptomatic biliary colic with a picture resembling a parasitic worm.
